# Secondhand Smoke Exposure Among University Students in the United Arab Emirates: Knowledge as a Correlate of Protective Behavior—A Cross-Sectional Study

**DOI:** 10.3390/ijerph23081008

**Published:** 2026-07-31

**Authors:** Tania Santina, Marwa Alahmed, Aiysha Faraj, Asma Arif, Jawaher Muhammed, Shayma Haidar, Wedad Almuaini, Nour Kawam, Abdallah Saab

**Affiliations:** 1 Faculty of Health Sciences, Higher Colleges of Technology, Sharjah P.O. Box 7947, United Arab Emirates; 2 Faculty of Public Health, Lebanese University, Saida P.O. Box 0714, Lebanon; 3 Faculty of Medicine, Beirut Arab University, Beirut P.O. Box 11-5020, Lebanon; nour-kawam@hotmail.com (N.K.); abdallahysaab@gmail.com (A.S.)

**Keywords:** environmental health, health behavior, secondhand smoke exposure, tobacco control, United Arab Emirates, university students

## Abstract

**Highlights:**

**Public health relevance—How does this work relate to a public health issue?**

- Secondhand smoke exposure (SHSE) remains a significant environmental health concern among university students in the United Arab Emirates, particularly in shared public and institutional settings.
- Persistent exposure despite existing smoke-free policies highlights ongoing challenges in reducing involuntary tobacco smoke exposure in socially embedded environments.

**Public health significance—Why is this work of significance to public health?**

- This study identifies a moderate-to-strong association between SHSE knowledge and protective behavior (R^2^ = 0.49), indicating that awareness is an important correlate of health-protective action.
- Knowledge was a moderate-to-strong correlate of protective behavior (r = 0.700); 44.2% of students demonstrated good protective behavior and 26.9% demonstrated poor protective behavior, indicating that unmeasured contextual and social factors likely still shape whether awareness translates into action for a meaningful subgroup.

**Public health implications—What are the key implications or messages for practitioners, policy makers and/or researchers in public health?**

- The findings support the need for strengthened enforcement of smoke-free policies and improved environmental controls in public and university settings.
- Integrated, culturally appropriate interventions addressing behavioral, social, and environmental factors are required to enhance protective health behaviors and reduce SHSE exposure.

**Abstract:**

Secondhand smoke exposure (SHSE) remains a major environmental health risk among university students, particularly in socially shared settings. In the United Arab Emirates, culturally embedded practices such as shisha and midwakh use, combined with variable policy enforcement, sustain persistent exposure. This cross-sectional study examined SHSE-related knowledge and protective health behaviors among 360 university students and their sociodemographic correlates, using a self-report questionnaire with documented internal consistency in comparable populations. Data were analyzed using SPSS version 28. SHSE was highly prevalent, occurring mainly in public and university settings; knowledge was moderate and protective behavior varied. Knowledge emerged as a moderate-to-strong correlate of protective behavior (r = 0.70, *p* < 0.001, R^2^ = 0.49). Age, employment, marital status, and number of children were associated with knowledge; age, gender, marital status, and income, but not employment, remained associated with behavior after adjusting for knowledge. Confirmatory factor analysis showed poor absolute fit for both the one- and two-factor solutions, and the behavioral-response factor did not satisfy the Fornell–Larcker criterion (√AVE = 0.750 < latent r = 0.836), providing only partial support for the empirical distinctiveness of the two constructs. Bootstrapped mediation analysis shows that knowledge partially mediated the employment–behavior association (~62% of the total effect); employment status was not, however, independently associated with behavior once knowledge was included, so this pathway is reported as hypothesis-generating. Still, more than a quarter of participants reported poor protective behavior, suggesting that unmeasured social and environmental factors, and possible overlap between the two constructs, may influence this relationship. Findings support integrated interventions combining health education, policy enforcement, and culturally tailored strategies.

## 1. Introduction

Secondhand smoke exposure (SHSE), also referred to as environmental tobacco smoke, is a major environmental health concern and a leading preventable risk factor for non-communicable diseases [1,2]. It occurs when non-smokers involuntarily inhale smoke emitted from burning tobacco products and exhaled by active smokers. No safe level of exposure has been established, and even brief exposure can result in immediate and long-term adverse health effects [1,3]. As such, SHSE represents a critical environmental determinant of population health and a priority area for tobacco control and disease prevention efforts.

Epidemiological evidence consistently shows increased risks of ischemic heart disease (RR = 1.27, 95% CI: 1.10–1.48), stroke (RR = 1.35, 95% CI: 1.22–1.50) [4], and lung cancer (RR = 1.24, 95% CI: 1.16–1.32) [5] among non-smokers exposed to SHSE. Beyond these health effects, tobacco use and SHSE impose a substantial economic burden, with global costs exceeding USD 1.4 trillion annually through healthcare expenditure and productivity losses [6]. Collectively, this evidence supports the relevance of SHSE as a priority for environmental public health and tobacco-control efforts.

Despite strong international consensus regarding SHSE harms, exposure patterns vary across regions, reflecting differences in cultural practices, tobacco product preferences, and regulatory environments [1,7]. In Gulf countries, tobacco consumption often occurs in social and recreational settings, frequently involving non-cigarette products such as waterpipe (shisha) and midwakh (dokha), which may increase opportunities for involuntary SHSE [7,8]. The growing availability of alternative tobacco products, including waterpipe and electronic cigarettes, has further complicated tobacco control efforts in the region [1,9].

The United Arab Emirates (UAE) provides a particularly relevant context for examining SHSE as an environmental health priority within a rapidly developing and socially dynamic setting. Tobacco consumption in the UAE includes both conventional cigarettes and traditional forms such as shisha and midwakh, which are commonly used in social environments and reflect established cultural practices [10,11]. These patterns may contribute to continued SHSE, particularly among young adults in shared public and institutional settings. The UAE has implemented comprehensive tobacco control policies and public health initiatives, including smoke-free regulations. Nevertheless, ongoing exposure in certain settings highlights the importance of further strengthening implementation and awareness efforts to enhance population health protection [6,12].

Tobacco use remains prevalent, with studies reporting smoking rates ranging from approximately 15% to 42%, particularly among university students [10,11,13]. Evidence also suggests that many non-smokers experience SHSE for up to 1–5 h per week in social or household environments [11]. National data indicate that SHSE accounted for 8.45% of all deaths in 2021 [14].

Emissions studies further demonstrate that indoor waterpipe smoking can generate carbon monoxide levels up to five times higher than cigarette smoke, with pollutants detectable several meters from the smoking source [15,16]. The continued use of shisha and midwakh in shared social environments may therefore sustain SHSE, despite the implementation of smoke-free policies. Population-based findings from the National Mutaba’ah Study further indicate continued SHSE in both domestic and public settings [17].

Previous studies in the UAE have primarily focused on tobacco use prevalence [10,13,18,19,20], rather than comprehensively examining SHSE-related knowledge, preventive behaviors, or determinants of protective responses, particularly among non-smoking university students.

To better understand the relationship between knowledge and protective health behavior toward SHSE, this study is conceptually informed by Health Belief Model [21]. This model posits that individuals are more likely to adopt protective health behaviors when they perceive a health threat as serious, recognize their susceptibility, and believe that taking action will reduce risk [22]. In the context of SHSE, knowledge of health risks can influence perceived severity and susceptibility, thereby motivating avoidance behaviors.

However, behavioral responses may also be influenced by measured exposure-related and contextual factors, including smoking status, tobacco product use, SHSE in the past 30 days, exposure frequency, and primary exposure locations such as cafés/restaurants, home, public places, university, and other settings. In addition, behavioral-response items captured protective actions and tolerance-related responses, such as leaving smoky areas, asking someone not to smoke nearby, and attitudes toward smoking in open spaces.

By applying a theoretical lens, this study provides a deeper understanding of how knowledge translates into protective behaviors in real-world social environments, which is essential for developing effective tobacco control strategies and strengthening smoke-free environments. Therefore, this study aims to examine knowledge and protective health behaviors related to SHSE, and to identify their determinants. This study contributes to the limited body of research in the UAE by simultaneously examining knowledge, protective health behavior, and their determinants related to SHSE within a unified analytical framework, thereby advancing behavioral epidemiology research on environmental tobacco exposure in the region. The findings provide context-specific evidence that may inform targeted tobacco-control interventions and smoke-free policy implementation for addressing environmental tobacco exposure among university students in the UAE.

## 2. Materials and Methods

### 2.1. Study Design and Settings

This cross-sectional observational study was conducted involving undergraduate and postgraduate students recruited from public and private universities in the UAE. The study followed the Strengthening the Reporting of Observational Studies in Epidemiology (STROBE) guidelines for observational research [23]. The completed STROBE checklist for the study is provided in Supplementary Table S1.

### 2.2. Ethics Statement

Ethical approval was obtained from the Higher Colleges of Technology Research Ethics Integrity Committee (Approval No. REIC2025-CAP53) on 23 April 2025. All procedures complied with the Declaration of Helsinki [24]. Participants received an electronic information sheet describing the study purpose, confidentiality protections, and voluntary nature of participation. Informed electronic consent was obtained before participants accessed the survey. Participation was voluntary, no incentives were offered, and participants could withdraw at any time without penalty. No personally identifiable information was collected. Data were stored on a password-protected institutional server with restricted access.

### 2.3. Participants and Sample Size

Eligible participants were undergraduate or postgraduate students aged 18 years or older, enrolled in universities across the UAE at the time of data collection, able to read and understand English, and willing to provide informed electronic consent. Both Emirati and expatriate students from diverse academic disciplines were eligible, reflecting the multicultural composition of the UAE higher-education system. Participants were recruited through universities located across the UAE.

A non-probability convenience sampling strategy was used due to logistical constraints and institutional recruitment permissions. Students were recruited through institutional email systems, university learning platforms, and QR-coded posters placed in common student areas such as cafeterias and student lounges.

The sample size was determined based on the study’s primary analytic objective: identifying determinants of SHSE-related knowledge and protective behavioral responses using multiple linear regression. A priori power analysis conducted using G*Power version 3.1 (Heinrich Heine University, Düsseldorf, Germany) [25] indicated that a minimum sample size of 300 participants was required, assuming a moderate effect size (f^2^ = 0.15), an alpha level of 0.05, and statistical power of 0.95 [26]. To account for incomplete responses, the recruitment target was increased to 360.

Conventional sample-size formulas presuppose probability sampling, an assumption not satisfied by the convenience sampling design employed in this study; the resulting calculation therefore reflects the nominal statistical precision of the regression analyses rather than protection against selection bias. Given the study’s additional descriptive aim of estimating the prevalence of SHSE-related knowledge, protective behavior, and exposure, a complementary calculation for a single proportion (worst-case *p* = 0.5, 95% confidence level, ±5% precision) was performed, yielding a target of approximately 384 participants. The achieved sample (*N* = 360) exceeded the regression-based threshold but fell short of this descriptive target; accordingly, the nominal sampling precision of the prevalence estimates is marginally wider than ±5%, whereas the non-probability sampling design introduces a structural limitation to representativeness and generalizability that precision adjustments alone cannot resolve.

### 2.4. Measures

Data were collected using a structured self-administered online questionnaire administered through Microsoft Forms (Microsoft Corporation, Redmond, WA, USA).

#### 2.4.1. Demographic and Exposure Variables

Thirteen demographic and exposure variables were collected: age, gender, academic level, nationality, academic discipline, income level, employment status, smoking status, type of tobacco used, exposure to secondhand smoke within the past 30 days, exposure frequency, and primary exposure locations (e.g., cafés, dormitories, and public spaces).

#### 2.4.2. SHSE Knowledge

SHSE knowledge was assessed using nine items developed by Sun and Frederic [27], measuring awareness of the harmful components of secondhand smoke and its associated health risks. Responses were recorded on a five-point Likert scale (0 = strongly disagree to 4 = strongly agree), with a “don’t know” option included. Composite scores ranged from 0 to 36, with higher scores indicating greater knowledge, and were categorized as poor (<18), satisfactory (18–27), or good (>27), consistent with the original instrument [27]. The original study reported good internal consistency for the scale (Cronbach’s α = 0.83).

#### 2.4.3. Behavioral Responses to SHSE

Behavioral responses were assessed using nine items from the same instrument [27], comprising protective behaviors (e.g., leaving smoky areas, asking others not to smoke) and tolerance indicators (e.g., acceptance of smoking in open spaces). Three items were reverse-coded. Composite scores ranged from 0 to 36, with higher scores reflecting stronger avoidance behaviors and lower SHSE tolerance, and were categorised as poor (<18), satisfactory (18–27), or good (>27), consistent with the original instrument [27]. The original study reported good internal consistency for the scale (Cronbach’s α = 0.85).

### 2.5. Data Collection Procedure

Prior to main data collection, the questionnaire was pilot-tested with 36 students, (~10% of the target sample), to assess item clarity and contextual appropriateness. Minor refinements were made based on participant feedback. Internal consistency was good for both the SHSE knowledge scale (Cronbach’s α = 0.86) and the behavioral response scale (Cronbach’s α = 0.87). These coefficients were interpreted as indicators of internal consistency reliability and not as evidence of construct validity in the UAE university population. Pilot participants were not invited to take part in the main survey and are not included in the *N* = 360 analytic sample reported below. Main data collection was conducted from April to May 2025. Participants reviewed the electronic information sheet, confirmed eligibility, and provided informed consent before accessing the questionnaire. The survey required approximately 10–15 min to complete.

### 2.6. Data Analysis

Data were exported from Microsoft Forms to Microsoft Excel 365 version 16.0 (Microsoft Corporation, Redmond, WA, USA) for initial cleaning and subsequently analyzed using IBM SPSS Statistics version 28 (IBM Corp., Armonk, NY, USA). Of the 369 students who accessed the survey, seven did not submit a response and two submitted responses containing no item-level data; these nine records were excluded, yielding a final analytic sample of 360 participants. Potential duplicate submissions were screened by checking for records sharing an identical combination of submission timestamp and response pattern; no records meeting this criterion were identified. For each of the two nine-item composite scales, a single missing item was replaced using the respondent’s mean across the completed items on that scale. Scale scores with two or more missing items were excluded from analyses involving that scale. Ten participants had one missing item on one or both scales and were retained in the composite-score analyses (*N* = 360). Item-level imputation was not performed for the confirmatory factor analysis; therefore, the CFA was restricted to participants with complete responses to all 18 items (*n* = 350). Missing monthly income data (*n* = 85) were handled separately using sample-mean substitution for regression analyses only. Sample-mean substitution preserves the analytic sample but attenuates the variance of the imputed variable and may understate its standard error; because monthly income was missing for 23.6% of participants and was retained as a statistically significant covariate in the behavioral-response model, this procedure is acknowledged as a limitation in Section 4.2 and the income coefficient should be interpreted with corresponding caution.

Descriptive statistics were used to summarize demographic characteristics and study variables. Independent samples t-tests were used for binary variables and one-way analysis of variance (ANOVA) for variables with more than two categories. Levene’s test was applied to assess homogeneity of variance assumptions. Pearson correlation coefficients were calculated to examine associations between knowledge and behavior scores. For the regression analyses described below, missing data on number of children (*n* = 298, asked only of participants with children) were recoded as zero, consistent with the skip-pattern design of this item. In Table 1, participants with unreported monthly income are shown separately as “Not reported” rather than assigned to an income band, because sample-mean substitution does not correspond to an observed category. The number-of-children recoding and income substitution described above retained the full analytic sample (*N* = 360) in the regression models rather than reducing it by listwise deletion. Religion was not included in the reported multivariable regression models because only one participant belonged to the comparison category; any coefficient would have been effectively determined by a single observation and would not provide a stable or generalizable estimate. Multiple linear regression was first performed to identify determinants of SHSE knowledge scores, with eight prespecified sociodemographic and exposure-related variables (age, gender, education level, employment status, marital status, number of children, nationality, and monthly income) entered simultaneously. For protective behavioral responses, hierarchical multiple linear regression was conducted by entering the same eight covariates in Model 1 and adding the SHSE knowledge score in Model 2. Backward elimination (removal criterion *p* > 0.05) was subsequently applied to the complete candidate models to derive parsimonious reduced models. The complete pre-elimination models reported in Table 2 and Table 3 provide the primary adjusted estimates, whereas the backward-reduced models, reported in Supplementary Tables S2 and S3, are presented as secondary parsimonious summaries. Because variable selection and coefficient estimation were conducted in the same sample, the confidence intervals and *p*-values from the reduced models do not account for model-selection uncertainty. Given that protective behavioral responses toward SHSE are conceptually most relevant for non-smokers, the hierarchical regression model predicting behavioral responses was replicated in the non-smoker subsample (*n* = 265, 73.6%) as a sensitivity analysis. Results are reported in Supplementary Table S4. For every hierarchical model, R^2^, adjusted R^2^, the change in R^2^ between steps (ΔR^2^), and the F statistic with its degrees of freedom and *p*-value are reported for each step, together with the F-change test for the addition of the SHSE knowledge score. Standard regression assumptions were verified, including linearity, normality of residuals, homoscedasticity, and multicollinearity; the latter was assessed using variance inflation factors (VIF), with values below 5.0 considered acceptable. Linearity and homoscedasticity were assessed by visual inspection of standardized residual-versus-predicted-value scatterplots, normality of residuals by P-P plots, and influential observations by Cook’s distance, with values exceeding 1 considered potentially influential; no observation exceeded this threshold in either regression model. To examine whether SHSE knowledge mediated the association between employment status and behavioral responses, a bootstrapped mediation analysis was performed using the PROCESS macro version 4.2 (Hayes, Columbus, OH, USA) [28] with 5000 resamples and bias-corrected accelerated (BCa) 95% confidence intervals. To evaluate the discriminant validity of the knowledge and behavioral-response scales given their strong observed correlation, confirmatory factor analysis (CFA) was conducted on the 18 item-level responses (9 knowledge items, 9 behavioral-response items; complete-case *n* = 350) using maximum-likelihood estimation. This item-level complete-case sample (*n* = 350) is smaller than the *N* = 360 regression sample because the mean-imputation procedure, applied to the composite knowledge and behavior scores, was not extended to individual items for the item-level CFA; the ten participants with missing item-level responses were therefore excluded listwise from this analysis only. A two-factor model, in which items loaded on their respective hypothesized construct with the two factors allowed to correlate, was compared against a one-factor model in which all 18 items loaded on a single construct. Model fit was evaluated using the comparative fit index (CFI), Tucker-Lewis index (TLI), and root mean square error of approximation (RMSEA), with CFI/TLI ≥ 0.90 and RMSEA ≤ 0.08 considered indicative of acceptable fit [29]. The two models were compared using a chi-square difference test. Discriminant validity between the two latent factors was further assessed using the Fornell–Larcker criterion, comparing the square root of each factor’s average variance extracted (AVE) against the inter-factor correlation [30]. Statistical significance was defined as *p* < 0.05 (two-tailed).

## 3. Results

### 3.1. Sample Characteristics

Of the 369 students who accessed the survey, seven did not submit a response and two submitted responses containing no item-level data. These nine records were excluded, yielding a final analytic sample of 360 participants (completion rate: 97.6%). The mean age of participants was 22.2 years (SD = 2.1). Most participants were female (63.6%), single (83.6%), Muslim (99.7%), Emirati (95.3%), and enrolled in undergraduate programs (83.3%). Most were students who were not employed (70.0%). Monthly income below AED 5000 was reported by 42.5% of participants, whereas 23.6% did not report monthly income.

Regarding tobacco use, 73.6% of participants were non-smokers and 26.4% were current smokers. Among current smokers, poly-tobacco product use was the most frequently reported pattern (45.3%). Overall, 74.4% of participants reported SHSE in the preceding month, most frequently in public spaces (38.8%) and university settings (29.1%).

The mean SHSE knowledge score was 24.6 (SD = 10.7). Of the participants, 37.8% were classified as having good knowledge (>27), 42.8% satisfactory knowledge (18–27), and 19.4% poor knowledge (<18). The mean protective behavioral-response score was 23.4 (SD = 8.6); 44.2% of participants demonstrated good protective behavioral responses (>27), 28.9% satisfactory responses (18–27), and 26.9% poor responses (<18). Sample characteristics are summarized in Table 1. Detailed item-level results for the SHSE knowledge and protective behavioral-response scales are provided in Supplementary Tables S5 and S6, respectively.

### 3.2. Association Between Knowledge and Protective Behavioral-Responses Toward SHSE

**Figure 1:**
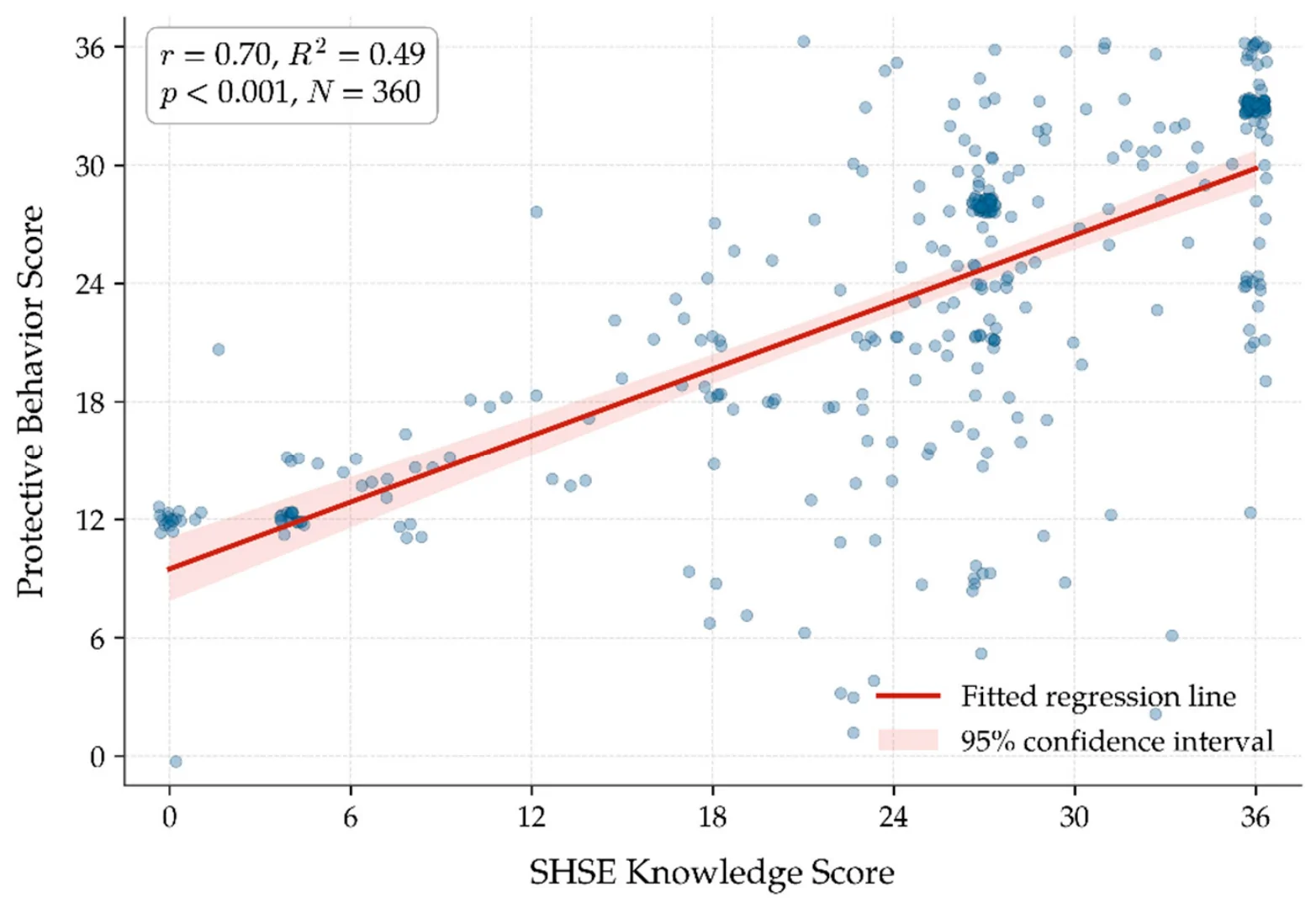
Association between SHSE knowledge and protective behavioral-response scores. Blue dots represent individual participants’ paired SHSE knowledge and protective behavioral-response scores. Jittered and semi-transparency were applied to reduce overplotting of identical score combinations. The solid red line represents the fitted linear regression line, and the shaded area represents the 95% confidence interval. SHSE, secondhand smoke exposure.

Pearson’s correlation analysis revealed a moderate-to-strong positive association between SHSE knowledge and protective behavioral-response scores (*r* = 0.700, *p* < 0.001). Simple linear regression further showed that SHSE knowledge was significantly associated with protective behavioral responses, accounting for approximately 49% of the variance in behavioral-response scores (*R*^2^ = 0.489, *F*(1, 358) = 343.07, *p* < 0.001). Each one-point increase in the SHSE knowledge score was associated with a 0.565-point increase in the protective behavioral-response score (*B* = 0.565, β = 0.700, *p* < 0.001). Although these findings indicate a moderate-to-strong association between the two measures in this sample, interpretation should consider the potential construct overlap examined in the measurement analyses below and discussed in Section 4.2. The association between SHSE knowledge and protective behavioral-response scores is illustrated in Figure 1.

The two-factor model provided a significantly better relative fit than the one-factor model (two-factor model: χ^2^(134) = 1588.6, CFI = 0.839, TLI = 0.816, RMSEA = 0.176; one-factor model: χ^2^(135) = 2489.5, CFI = 0.740, TLI = 0.705, RMSEA = 0.224; Δχ^2^(1) = 900.9, *p* < 0.001). However, the absolute fit of the two-factor model remained below the prespecified conventional thresholds, and the latent Knowledge–Behavior correlation was high (*r* = 0.836).

The Fornell–Larcker criterion was satisfied for the Knowledge factor (√AVE = 0.924 > 0.836) but not for the Behavior factor (√AVE = 0.750 < 0.836). Weak measurement performance was particularly evident in the three reverse-coded tolerance items, which had standardized loadings of 0.22, 0.30, and −0.18, whereas the six protective-action items showed strong loadings (0.86–0.95). Because both models fell short of the prespecified CFI/TLI ≥ 0.90 and RMSEA ≤ 0.08 thresholds, the latent factor correlation was high, and discriminant validity was not established for the Behavior factor, these findings provide only partial support for the distinction between the two constructs, given the suboptimal model fit, and do not exclude meaningful item-level overlap between the scales. The reverse-coded item with an unexpected-direction loading was rechecked against the original coding scheme; no coding error was identified, so the loading was interpreted as reflecting the item’s empirical performance in the present sample rather than a data-entry or reverse-scoring error.

### 3.3. Determinants of Knowledge Related to SHSE

Multiple linear regression was used to examine determinants of SHSE knowledge scores. The complete model included eight prespecified covariates and was statistically significant (F(8, 351) = 18.000, *p* < 0.001; adjusted R^2^ = 0.275). In this primary adjusted analysis, age was positively associated with knowledge score (B = 2.398, 95% CI: 0.811–3.984, β = 0.161, *p* = 0.003), whereas employment status (B = −5.085, 95% CI: −7.908 to −2.263, β = −0.219, *p* < 0.001), marital status (B = −4.134, 95% CI: −6.678 to −1.591, β = −0.198, *p* = 0.002), and number of children (B = −4.059, 95% CI: −5.751 to −2.366, β = −0.280, *p* < 0.001) were negatively associated with knowledge. Gender, education level, nationality, and monthly income were not independently associated with knowledge. VIF values ranged from 1.03 to 1.89, indicating no problematic multicollinearity. Complete estimates are reported in Table 2.

Backward elimination produced a reduced model containing the same four associated variables (F(4, 355) = 34.797, *p* < 0.001; adjusted R^2^ = 0.274). The reduced-model estimates were closely aligned with the complete-model estimates, including age (B = 2.382), employment status (B = −5.493), marital status (B = −4.377), and number of children (B = −4.124). Supplementary Table S2 presents this reduced model as a secondary parsimonious summary; interpretation is based on the complete model reported in Table 2, and the confidence intervals and *p*-values in the reduced model do not account for model-selection uncertainty.

### 3.4. Determinants of Behavior Responses Related to SHSE

Hierarchical multiple linear regression examined determinants of protective behavioral responses. The same eight covariates included in the knowledge model were entered in Model 1, and SHSE knowledge was added in Model 2. In the complete Model 1, age, gender, employment status, marital status, and monthly income were independently associated with behavioral-response score (F(8, 351) = 18.873, *p* < 0.001; R^2^ = 0.301; adjusted R^2^ = 0.285). After knowledge was added, the complete Model 2 was statistically significant (F(9, 350) = 54.252, *p* < 0.001; adjusted R^2^ = 0.572), and the addition of the SHSE knowledge score produced a significant increment in explained variance (R^2^ = 0.582; ΔR^2^ = 0.282; F-change (1, 350) = 236.144, *p* < 0.001). Knowledge was the strongest correlate (B = 0.509, 95% CI: 0.444–0.575, β = 0.630, *p* < 0.001). Age (B = 1.479, 95% CI: 0.482–2.476, β = 0.123, *p* = 0.004) and gender (B = 3.817, 95% CI: 2.533–5.101, β = 0.213, *p* < 0.001) were positively associated with behavioral-response score, whereas marital status (B = −2.082, 95% CI: −3.684 to −0.480, β = −0.123, *p* = 0.011) and monthly income (B = −1.813, 95% CI: −2.645 to −0.982, β = −0.197, *p* < 0.001) were negatively associated. Education level, employment status, number of children, and nationality were not independently associated after knowledge was included. Complete estimates are reported in Table 3.

Backward elimination produced a reduced model retaining age, gender, marital status, monthly income, and knowledge score (F(5, 354) = 96.488, *p* < 0.001; adjusted R^2^ = 0.571). The estimates were closely aligned with those from the complete Model 2, including the knowledge coefficient (B = 0.502 versus 0.509 in the complete model). Supplementary Table S3 presents the reduced model as a secondary parsimonious summary; interpretation is based on the complete hierarchical model reported in Table 3, and the confidence intervals and *p*-values in the reduced model do not account for model-selection uncertainty.

Employment status was associated with behavioral response in the complete Model 1 but attenuated after knowledge was added in Model 2. This attenuation was further examined in the mediation analysis reported below. VIF values in the complete Model 2 ranged from 1.03 to 1.95, indicating no problematic multicollinearity.

A sensitivity analysis restricted to non-smokers (*n* = 265) yielded results consistent with the primary analysis. The hierarchical model was replicated in this subsample: Model 1, containing the sociodemographic covariates only, was statistically significant (F(8, 256) = 12.189, *p* < 0.001; R^2^ = 0.276; adjusted R^2^ = 0.253), and the addition of the SHSE knowledge score in Model 2 produced a significant increment in explained variance (F(9, 255) = 50.077, *p* < 0.001; R^2^ = 0.639; adjusted R^2^ = 0.626; ΔR^2^ = 0.363; F-change(1, 255) = 256.035, *p* < 0.001). Knowledge remained the strongest correlate of behavioral response (B = 0.508, 95% CI: 0.445–0.570, β = 0.684, *p* < 0.001), supporting the robustness of the principal finding. Full coefficient estimates and model-summary statistics for each step are reported in Supplementary Table S4.

The bootstrapped mediation analysis (5000 resamples; *N* = 360) showed significant paths from employment status to SHSE knowledge (path *a*: *B* = −7.205, *p* < 0.001) and from knowledge to behavioral responses, controlling for employment status (path *b*: B = 0.534, *p* < 0.001). The indirect effect (−3.848; 95% BCa CI [−5.199, −2.573]) accounted for ~62% of the total effect (total effect, path *c*: *B* = −6.196, *p* < 0.001), with a significant direct effect remaining (path *c*′: *B* = −2.348, *p* = 0.002), indicating that knowledge partially, rather than fully, mediates the employment–behavior association. Detailed path estimates are provided in Supplementary Table S7.

## 4. Discussion

This study examined SHSE-related knowledge and protective behavioral responses among university students in the UAE and identified SHSE knowledge as a moderate-to-strong correlate of protective behavior. Interpreted through the Health Belief Model [21,22], this finding is consistent with the premise that awareness of health risks contributes to perceived susceptibility and severity, thereby supporting protective action. The mediation analysis further suggests that SHSE knowledge partially mediates the association between employment status and behavioral responses, with a significant direct effect remaining. These findings should nonetheless be interpreted as evidence of association rather than causation, given the cross-sectional design, and the observed relationship may be influenced by additional contextual factors that were not directly measured.

Despite the existence of national tobacco-control regulations and policies [1,12], SHSE remained common in this sample, particularly in public and university settings. This persistence is consistent with regional evidence that tobacco control in Gulf countries is shaped not only by regulatory frameworks but also by cultural practices and the social acceptability of tobacco use [9,31]. In the UAE specifically, the continued popularity of shisha and midwakh in shared social settings likely sustains exposure [10,11], an effect compounded by the higher carbon-monoxide output and broader pollutant dispersion of waterpipe relative to cigarette smoking [15,16]. Previous UAE-based studies have similarly documented ongoing exposure in public, domestic, and institutional settings despite formal smoke-free policies [10,11,17]. A similar enforcement-dependent pattern has been documented elsewhere in the Gulf Cooperation Council, indicating that this is a regional rather than a purely UAE-specific phenomenon: in Saudi Arabia, a government-mandated tobacco sales ban within the holy cities of Mecca and Medina achieved only partial retailer compliance (75%) and carried no penalty for individual smoking in public, allowing waterpipe and cigarette use to persist through smuggled or self-supplied products [32]; and at Qatar University, strong overall support for a tobacco-free campus policy (75.5% among students) was measurably weaker among current tobacco users, indicating that social acceptability, rather than policy adoption alone, governs real-world compliance [33]. A structurally similar pattern, though arising in a geographically and culturally distinct setting, is evident in rapidly urbanizing parts of East Asia: a comprehensive, strictly enforced smoke-free ordinance in Shenzhen, China, was associated with measurable reductions in stroke incidence following its introduction [34], whereas a comparably legislated ordinance in nearby Qingdao showed markedly uneven compliance across enforcement agencies, including persistent designated-smoking areas that the policy explicitly prohibits [35], a divergence further corroborated by recent global reviews of smoke-free policy compliance [36,37]. Taken together, this evidence, convergent across the Gulf region and structurally consistent even in a divergent cultural context, reinforces the conclusion that knowledge-based interventions alone are unlikely to be sufficient in the UAE: strengthening enforcement, monitoring compliance at the point of service, and addressing the social acceptability of shisha and midwakh use in shared social settings are necessary complements to health education in this cultural context [10,11].

Knowledge emerged as the strongest correlate of protective behavioral responses in this study, consistent with prior research linking greater SHSE-related awareness to stronger avoidance behavior [27]. Two considerations nonetheless warrant caution in interpreting the magnitude of this association. First, the knowledge and behavioral-response scales were derived from the same source instrument [27] and were completed by the same respondents using the same self-report response format. Part of the observed association may therefore reflect common-method variance associated with the shared source and measurement method [38], in addition to substantive or measurement overlap between the constructs. This is presented as a potential limitation and not as evidence that common-method bias was definitively present. Second, the Health Belief Model itself holds that perceived susceptibility and severity are necessary but not sufficient for action: modifying factors such as social norms, self-efficacy, and environmental opportunity also shape whether individuals act on their knowledge [21,22], consistent with the subjective-norm and perceived-behavioral-control constructs of the Theory of Planned Behavior [39]. Accordingly, although 44.2% of participants demonstrated good protective behavior, more than a quarter (26.9%) demonstrated poor protective behavior despite the overall knowledge–behavior association; this substantial minority should be interpreted in relation to, rather than in contradiction with, the moderate-to-strong knowledge–behavior association observed here. Protective actions such as leaving smoke-exposed environments, asking others not to smoke, or avoiding exposure altogether may be constrained by interpersonal expectations, the social acceptability of smoking, perceived authority to intervene, and the availability of smoke-free alternatives, factors that were not directly measured in this study and may represent important unmeasured moderators of the knowledge–behavior relationship. These findings are therefore best interpreted as showing that SHSE knowledge is an important correlate of protective behavior for most students, while sustained behavioral change for the remaining minority is likely to require complementary environmental, institutional, and policy-level support, underscoring the need for multi-level interventions that address social and environmental determinants of behavior alongside individual awareness.

The sociodemographic determinants identified in this study provide further insight into variation in SHSE-related knowledge and behavior. Older and non-employed students reported higher knowledge scores than younger or employed students. One possible explanation is that greater employment commitments may be associated with less frequent campus attendance [40], which could limit exposure to campus-based health-promotion information. Because employment hours, attendance, and such exposure were not measured here, this mechanism remains plausible but untested. That study was conducted in a North American university setting, so the transportability of the employment–attendance association to UAE campuses is assumed rather than demonstrated.

Students with more children also reported lower knowledge scores, independent of marital status; this may reflect competing caregiving demands that reduce engagement with campus-based health-promotion activities, although this interpretation is exploratory given the cross-sectional design. Marital status was similarly associated with both knowledge and behavioral responses, with married, divorced, and widowed students scoring lower than single students; given the small size of these subgroups (e.g., *n* = 1 widowed), this finding should be considered exploratory. For behavioral responses specifically, female students reported notably stronger protective responses than male students, the largest sociodemographic effect on behavior after knowledge itself; older students and those with lower monthly income also reported stronger protective responses, in addition to the single-student pattern already noted for marital status. The income association may reflect that students with greater financial independence have more autonomy to leave or avoid smoke-exposed settings (e.g., choosing where to eat, live, or socialize), whereas the age association may reflect cumulative social experience or maturation in assertiveness toward unwanted smoke exposure. Neither education level nor nationality was independently associated with knowledge or behavioral responses in the corrected complete models; the postgraduate and expatriate-nationality associations observed in preliminary analyses were not reproduced after the near-singleton Religion covariate was removed and are not interpreted as substantive. The bootstrapped mediation analysis indicated that SHSE knowledge partially mediated the association between employment status and behavioral responses (accounting for approximately 62% of the total effect), a pattern theoretically consistent with the Health Belief Model [21,22], though not interpretable causally given the cross-sectional design.

### 4.1. Public Health Implications

From an environmental public health perspective, these findings support integrated tobacco-control strategies that combine educational, environmental, and policy-level approaches. Because SHSE was reported most frequently in public spaces and university settings, enforcement of smoke-free regulations should be strengthened in these environments to reduce involuntary exposure and improve compliance with existing tobacco-control policies [1,6,12]. Given the prominence of shisha and midwakh use in the UAE context, health-promotion initiatives should explicitly address waterpipe- and midwakh-related social norms and misconceptions rather than relying exclusively on cigarette-focused messaging [10,11,15]. The moderate-to-strong association observed between SHSE knowledge and protective behavior indicates that educational interventions remain important; however, they should be complemented by strategies that address social and environmental barriers to protective action, such as skills-based training to strengthen confidence in avoiding smoke-exposed settings, peer-led initiatives that challenge normative acceptance of smoking in shared environments, and institutional measures that facilitate adherence to smoke-free policies [21,22]. Together, these findings support university-based tobacco-control strategies that recognize knowledge as an important correlate of protective behavior while also addressing the broader contextual factors that shape behavioral responses.

These findings contribute to the behavioral epidemiology literature by demonstrating a moderate-to-strong association between SHSE knowledge and protective behavioral responses among university students in the UAE, while underscoring the importance of distinguishing statistical association from causal inference and of accounting for measurement overlap and unmeasured contextual influences when interpreting knowledge–behavior relationships. Future research should adopt longitudinal designs, incorporate objective measures of SHSE exposure, formally evaluate discriminant validity between the knowledge and behavioral-response constructs, and examine social, institutional, and environmental moderators that may shape protective behavior.

### 4.2. Strengths and Limitations

This study has several strengths. Participants were recruited from multiple university settings across the UAE, allowing SHSE-related knowledge and behavioral responses to be examined across more than one institutional context. The study also employed an instrument with documented internal consistency in comparable populations and an analytical approach that simultaneously examined knowledge and behavioral responses while adjusting for multiple sociodemographic variables. Furthermore, the sensitivity analysis restricted to non-smokers yielded findings consistent with the primary analysis, supporting the robustness of the observed knowledge–behavior association.

A few methodological considerations should be noted. First, the cross-sectional design precludes causal inference regarding the relationship between SHSE knowledge and behavioral responses, including the mediation pathway identified in the analysis. Second, the use of scale-item imputation and the predominance of Emirati participants may limit generalizability to the broader UAE university student population and introduce selection bias. Recruitment combined participants from public institutions that, by policy, enroll Emirati nationals almost exclusively and whose student body is approximately 63% female, together with other private institutions; this combination likely explains much of the sample’s high proportion of Emirati (95.3%) and female (63.6%) participants. Nationally, the UAE higher-education student body is more demographically mixed: women comprised approximately 54% of new higher-education enrollments in the 2024–2025 academic year [41], and because most UAE tertiary students attend private institutions in which Emirati nationals are a minority, the national student body includes a substantially larger expatriate share than observed here [42]. The present findings should therefore be interpreted as most representative of contexts similar to federal and mixed public/private institutions rather than the full diversity of the UAE higher-education population, particularly with respect to nationality.

Emirate-level residence or campus information was not collected, so the geographic distribution of the sample across the seven emirates could not be verified and may not be uniform. Recruitment through cafeterias and student lounges may have under-represented both current smokers and students highly conscious of SHSE, since these spaces are common social settings where smoking is frequently observed. This recruitment channel could plausibly bias the sample in either direction: students who smoke or who socialize in smoking-permissive areas may have been more likely to encounter the recruitment materials and enroll, which could inflate observed smoking prevalence and SHSE exposure estimates relative to the broader student population, whereas students who actively avoid these spaces because of smoke aversion may have been under-represented, which would understate the range of protective behavior present in that population. Because the direction and magnitude of this bias cannot be determined from the present data, the prevalence and behavioral estimates reported here should be treated as descriptive of this sample rather than as representative population estimates. Third, all measures were self-reported and may therefore be subject to recall and social desirability biases. Fourth, although the instrument demonstrated good internal consistency in this sample, it was originally developed in a different cultural context and formal cross-cultural validation was not undertaken. Fifth, smokers and non-smokers were both included in the primary regression models predicting behavioral responses; although a sensitivity analysis restricted to non-smokers yielded consistent results, protective behavioral responses to SHSE are most directly interpretable among non-smokers, and this should be borne in mind when interpreting the primary behavioral-response models. The backward-elimination models were used only as secondary parsimonious summaries; because selection and estimation were performed in the same sample, their inferential statistics do not account for model-selection uncertainty [43], and interpretation is based on the complete models reported in Supplementary Tables S8–S10. Finally, religion was not included in the reported regression models because the sample showed near-complete homogeneity for this variable, leaving the comparison category too small to yield a stable or meaningful estimate. Future research using probability-based or multi-site recruitment strategies should assess validity and measurement invariance in UAE populations.

One further point concerns the interpretation of the moderate-to-strong association between knowledge and behavioral responses. Although the scales were analyzed as conceptually distinct composite measures consistent with the source instrument [27], the observed inter-scale correlation indicates meaningful empirical proximity in this population. The two-factor CFA model fitted significantly better than the one-factor model; however, both models showed poor absolute fit, the latent correlation was high, and the Behavior factor did not meet the Fornell–Larcker criterion. The findings therefore provide only partial support for treating knowledge and behavioral responses as empirically distinct constructs in this sample. The weak reverse-coded tolerance items contributed to the low AVE of the Behavior factor and should be revised or replaced and re-evaluated in an independent, larger, and more diverse sample. Moreover, several potentially important determinants of protective behavior, including perceived behavioral control, social norms, self-efficacy, institutional enforcement, and availability of smoke-free environments, were not directly measured. These factors may moderate or mediate the relationship between knowledge and behavior and should be incorporated into future research. Longitudinal, multi-site, and mixed-methods studies incorporating objective measures of SHSE exposure would provide a more comprehensive understanding of the determinants of protective behavior among university students.

## 5. Conclusions

SHSE remains a significant environmental health concern among university students in the UAE, occurring mainly in public and university settings despite existing smoke-free regulations. SHSE knowledge was moderate and emerged as the strongest correlate of protective behavioral responses, consistent with the Health Belief Model premise that awareness underpins protective action. Nonetheless, more than a quarter of students reported poor protective behavior despite this association, indicating that unmeasured social, institutional, and environmental factors, together with possible construct overlap between the knowledge and behavior scales, likely shape whether awareness translates into sustained protective action for a meaningful subgroup. Reducing SHSE among UAE university students therefore likely requires strengthened enforcement of smoke-free policies in public spaces and university settings, health-promotion messaging that explicitly addresses shisha- and midwakh-related norms rather than cigarette use alone, and interventions targeting the social acceptability of smoking in shared spaces, delivered alongside continued knowledge-building efforts.

## Figures and Tables

**Table 1:** Sociodemographic characteristics and SHSE-related variables of the study sample.

Variable	Category	Frequency	(%)
Age	18–20 years	69	19.2
	21–23 years	216	60.0
	24–26 years	61	16.9
	≥27 years	14	3.9
Gender	Female	229	63.6
	Male	131	36.4
Marital Status	Single	301	83.6
	Married	44	12.2
	Divorced	14	3.9
	Widowed	1	0.3
Number of Children (*n* = 62)	1 child	27	43.5
	2 children	23	37.1
	≥3 children	12	19.4
Religion	Islam	359	99.7
	Other	1	0.3
Nationality	Emirati	343	95.3
	Expatriate	17	4.7
Education Level	Undergraduate	300	83.3
	Postgraduate	60	16.7
Employment Status	Student (Not Employed)	252	70.0
	Full-time Employed	59	16.4
	Part-time/Self-Employed	49	13.6
Monthly Income (AED)	<5000	153	42.5
	5000–10,000	61	16.9
	10,001–15,000	24	6.7
	>15,000	37	10.3
	Not reported	85	23.6
Smoking Status	Non-smoker	265	73.6
	Current smoker	95	26.4
Tobacco Products Used (*n* = 95)	Cigarettes only	12	12.6
	Midwakh only	14	14.7
	Shisha/Waterpipe only	17	17.9
	Poly-tobacco product use	43	45.3
	Vape only	1	1.1
	Not specified	8	8.4
SHSE in the Past Month	Not exposed	92	25.6
	Exposed	268	74.4
Primary SHSE Location (*n* = 268)	Cafés/restaurants	42	15.7
	Home	39	14.6
	Public spaces	104	38.8
	University	78	29.1
	Other	5	1.9
SHSE Knowledge Score	Poor (<18)	70	19.4
	Satisfactory (18–27)	154	42.8
	Good (>27)	136	37.8
Protective Behavioral-Response Score	Poor (<18)	97	26.9
	Satisfactory (18–27)	104	28.9
	Good (>27)	159	44.2

Note: SHSE, secondhand smoke exposure; AED, United Arab Emirates dirham. Unless otherwise specified, percentages were calculated using the full analytic sample (*N* = 360); percentages for subgroup variables were calculated using the corresponding subgroup denominator. SHSE Knowledge and protective behavioral-response scores ranged from 0 to 36 and were classified according to Sun and Frederic [27].

**Table 2:** Multiple linear regression model of determinants of SHSE knowledge scores (*N* = 360).

Independent Variable	B	95% CI for B	β	*p*-Value	VIF
Age	2.398	(0.811, 3.984)	0.161	0.003	1.46
Gender	−0.632	(−2.699, 1.434)	−0.029	0.548	1.11
Education Level	2.059	(−1.069, 5.187)	0.072	0.196	1.53
Employment Status	−5.085	(−7.908, −2.263)	−0.219	<0.001	1.88
Marital Status	−4.134	(−6.678, −1.591)	−0.198	0.002	1.89
Number of Children	−4.059	(−5.751, −2.366)	−0.280	<0.001	1.74
Nationality	1.581	(−2.799, 5.960)	0.032	0.478	1.03
Monthly Income	−1.253	(−2.585, 0.080)	−0.110	0.065	1.74

Note: B, unstandardized regression coefficient; CI, confidence interval; β, standardized regression coefficient; VIF, variance inflation factor. The complete pre-elimination model in Table 2 constitutes the primary adjusted analysis. Supplementary Table S2 presents the backward-reduced model as a secondary parsimonious summary; its confidence intervals and *p*-values do not account for variable-selection uncertainty.

**Table 3:** Multiple hierarchical linear regression model predicting protective behavioral-response scores toward SHSE (Model 2; *N* = 360).

Independent Variable	B	95% CI for B	β	*p*-Value	VIF
Age	1.479	(0.482, 2.476)	0.123	0.004	1.49
Gender	3.817	(2.533, 5.101)	0.213	<0.001	1.11
Education Level	1.413	(−0.534, 3.361)	0.061	0.154	1.54
Employment Status	−0.526	(−2.310, 1.258)	−0.028	0.563	1.95
Marital Status	−2.082	(−3.684, −0.480)	−0.123	0.011	1.94
Number of Children	0.803	(−0.281, 1.887)	0.068	0.146	1.85
Nationality	−0.713	(−3.435, 2.008)	−0.018	0.607	1.03
Monthly Income	−1.813	(−2.645, −0.982)	−0.197	<0.001	1.76
SHSE Knowledge Score	0.509	(0.444, 0.575)	0.630	<0.001	1.41

Note: B, unstandardized regression coefficient; CI, confidence interval; β, standardized regression coefficient; VIF, variance inflation factor. The complete pre-elimination hierarchical models in Table 3 constitute the primary adjusted analyses. Supplementary Table S3 presents the backward-reduced model as a secondary parsimonious summary; its confidence intervals and *p*-values do not account for variable-selection uncertainty.

## Data Availability

The datasets generated and analyzed during the present study, together with Supplementary Tables S1–S10, will be deposited in Zenodo and assigned a DOI upon acceptance, and are additionally available upon reasonable request from the corresponding author.

## Supplementary Materials

The following supporting information can be downloaded at: https://www.mdpi.com/article/10.3390/ijerph23081008/s1, Table S1: Completed STROBE checklist; Table S2: Knowledge model—backward-reduced model (secondary summary); Table S3: Behavioral-response model—backward-reduced model (secondary summary); Table S4: Hierarchical multiple linear regression predicting behavioral-response scores among non-smokers; Table S5: Participants’ responses to the secondhand smoke exposure knowledge items; Table S6: Participants’ behavioral responses toward secondhand smoke exposure; Table S7: Bootstrapped mediation analysis: employment status → SHSE knowledge → behavioral response; Table S8: Knowledge model—full pre-elimination model; Table S9: Behavioral-response model 1—full pre-elimination model without knowledge; Table S10: Behavioral-response model 2—full pre-elimination model with knowledge.

## Abbreviations

The following abbreviations are used in this manuscript:

SHSE	secondhand smoke exposure
UAE	United Arab Emirates
SD	Standard Deviation
VIF	Variance Inflation Factor
USD	United States Dollar
RR	Relative Risk
CI	Confidence Interval
STROBE	Strengthening the Reporting of Observational Studies in Epidemiology
CFA	Confirmatory Factor Analysis
CFI	Comparative Fit Index
TLI	Tucker-Lewis Index
RMSEA	Root Mean Square Error of Approximation
AVE	Average Variance Extracted
Cronbach’s α	Cronbach’s alpha (a measure of internal consistency reliability)
%	percentage
R^2^	coefficient of determination
Β	standardized regression coefficient
r	Pearson correlation coefficient
*p*	probability value (statistical significance)
