# Supplementary material for: Secondhand Smoke Exposure Among University Students in the United Arab Emirates: Knowledge as a Correlate of Protective Behavior—A Cross-Sectional Study
Source: Int J Environ Res Public Health. 2026 Jul 31;23(8):1008. doi: 10.3390/ijerph23081008 (PMC13513471; doi:10.3390/ijerph23081008)
Supplement: Supplementary file 1 [file ijerph-23-01008-s001.zip › ijerph-4271523-supplementary.pdf]

Table S1. Completed STROBE checklist. STROBE Statement — checklist of items that should be included in reports of cross-sectional studies. Adapted from the STROBE Statement (von Elm et al., 2007), publicly available for author use at www.strobe-statement.org. Page numbers refer to the pagination printed in the manuscript header.

Section/Topic	Item No.	Recommendation	Page No.
**Title and abstract**			
Title and abstract	1	(a) Indicate the study's design with a commonly used term in the title or the abstract	p. 1 (title)
		(b) Provide in the abstract an informative and balanced summary of what was done and what was found	pp. 1–2 (Abstract)
**Introduction**			
Background/rationale	2	Explain the scientific background and rationale for the investigation being reported	p. 2 (Sect. 1)
Objectives	3	State specific objectives, including any prespecified hypotheses	p. 3 (Sect. 1; no prespecified hypotheses)
**Methods**			
Study design	4	Present key elements of study design early in the paper	p. 4 (Sect. 2.1)
Setting	5	Describe the setting, locations, and relevant dates, including periods of recruitment, exposure, follow-up, and data collection	pp. 4–5 (Sect. 2.1; past-30-day exposure window; Apr–May 2025 data collection; no follow-up)
Participants	6	(a) Give the eligibility criteria, and the sources and methods of selection of participants	p. 4 (Sect. 2.3)
Variables	7	Clearly define all outcomes, exposures, predictors, potential confounders, and effect modifiers. Give diagnostic criteria, if applicable	p. 5 (Sects. 2.4.1–2.4.3); p. 8 (eight covariates; no effect modifiers)
Data sources/measurement	8*	For each variable of interest, give sources of data and details of methods of assessment (measurement). Describe comparability of assessment methods if there is more than one group	p. 5 (Sects. 2.4–2.5; measurement, pilot testing, survey administration)
Bias	9	Describe any efforts to address potential sources of bias	p. 4 (convenience sampling and precision caveat); pp. 7–9 (pilot testing, duplicate screening, missing-data rules); p. 14 (non-smoker sensitivity analysis)
Study size	10	Explain how the study size was arrived at	p. 4 (Sect. 2.3; G*Power and complementary proportion calculations)
Quantitative variables	11	Explain how quantitative variables were handled in the analyses. If applicable, describe which groupings were chosen and why	p. 5 (score construction, categories, and analytic recoding)
Statistical methods	12	(a) Describe all statistical methods, including those used to control for confounding	pp. 5–7 (Sect. 2.6)
		(b) Describe any methods used to examine subgroups and interactions	pp. 6–7 (non-smoker subgroup; no interaction analyses)
		(c) Explain how missing data were addressed	pp. 7–8 (item imputation, income substitution, skip-pattern recoding, complete-case CFA)
		(d) If applicable, describe analytical methods taking account of sampling strategy	N/A — convenience sample; no weighting or complex-sample analysis. Limitation noted pp. 5, 18
		(e) Describe any sensitivity analyses	pp. 7–11 (non-smoker sensitivity method); p. 14 (results)

Section/Topic	Item No.	Recommendation	Page No.
**Results**			
Participants	13*	(a) Report numbers of individuals at each stage of the study — e.g., numbers potentially eligible, examined for eligibility, confirmed eligible, included in the study, completing follow-up, and analyzed	p. 8 (survey access, exclusions, analytic sample); p. 11 (Sect. 3.1, completion rate)
		(b) Give reasons for non-participation at each stage	pp. 8, 11 (seven non-submissions; two records with no item-level data)
		(c) Consider use of a flow diagram	No flow diagram; numbers and exclusions reported in text (pp. 8, 11)
Descriptive data	14*	(a) Give characteristics of study participants (e.g., demographic, clinical, social) and information on exposures and potential confounders	pp. 9–10 (Table 1); p. 11 (Sect. 3.1)
		(b) Indicate number of participants with missing data for each variable of interest	pp. 8–10 (missing scale items, income, skip-pattern recoding, CFA complete cases, subgroup denominators)
Outcome data	15*	Report numbers of outcome events or summary measures	pp. 9–10 (Table 1); p. 11 (Sect. 3.1)
Main results	16	(a) Give unadjusted estimates and, if applicable, confounder-adjusted estimates and their precision (e.g., 95% confidence interval). Make clear which confounders were adjusted for and why they were included	p. 8 (prespecified adjustment covariates); pp. 10–11 (Tables 2–3, adjusted estimates and 95% CIs); p. 12 (unadjusted correlation/simple regression); pp. 13–14 (Sects. 3.3–3.4)
		(b) Report category boundaries when continuous variables were categorized	p. 7 (knowledge/behavior score thresholds); pp. 9–10 (Table 1: age, income, score categories)
		(c) If relevant, consider translating estimates of relative risk into absolute risk for a meaningful time period	N/A — linear regression outcomes, not risk ratios
Other analyses	17	Report other analyses done — e.g., analyses of subgroups and interactions, and sensitivity analyses	pp. 8–9 (methods: backward elimination, non-smoker subgroup, mediation, CFA); pp. 12–14 (results)
**Discussion**			
Key results	18	Summarize key results with reference to study objectives	pp. 14–15 (Sect. 4, opening paragraph)
Limitations	19	Discuss limitations of the study, taking into account sources of potential bias or imprecision. Discuss both direction and magnitude of any potential bias	pp. 17–19 (Sect. 4.2)
Interpretation	20	Give a cautious overall interpretation of results considering objectives, limitations, multiplicity of analyses, results from similar studies, and other relevant evidence	pp. 14–19 (Sects. 4–4.2)
Generalizability	21	Discuss the generalizability (external validity) of the study results	p. 18 (Sect. 4.2; convenience-sampling and representativeness discussion)
**Other information**			
Funding	22	Give the source of funding and the role of the funders for the present study and, if applicable, for the original study on which the present article is based	p. 20 (Funding statement)

** Give information separately for exposed and unexposed groups — here, for smokers and non-smokers — where applicable to this study.*

Supplementary Regression Models and Mediation Analysis (Tables S2–S4 and S7–S10)

Tables S2 and S4 present exploratory backward-elimination models; because variable selection and estimation were performed in the same sample, their nominal *p*-values and confidence intervals may be optimistic. Table S4 reports a sensitivity analysis replicating the hierarchical behavioral-response model among non-smokers ($n = 265$), and Table S7 presents the bootstrapped mediation analysis. The complete pre-elimination models in Tables S8–S10 provide the primary inferential basis: Table S8 corresponds to manuscript Table 2, while Tables S9 and S10 present Steps 1 and 2 of the hierarchical behavioral-response model, respectively, with Table S10 corresponding to manuscript Table 3. Religion was excluded from all multivariable models because only one participant belonged to the comparison category, precluding stable estimation. Unless otherwise specified, the analytic sample was $N = 360$.

Table S2. Knowledge model: backward-reduced model (secondary summary).

$F(4, 355) = 34.797$, $p < 0.001$; $R^2 = 0.282$; adjusted $R^2 = 0.274$; $N = 360$.

Independent variable	B	95% CI for B	β	*p*-Value	VIF
Age	2.382	(0.876, 3.888)	0.160	0.002	1.31
Employment status	−5.493	(−7.932, −3.054)	−0.236	<0.001	1.40
Marital status	−4.377	(−6.910, −1.843)	−0.209	0.001	1.87
Number of children	−4.124	(−5.798, −2.450)	−0.284	<0.001	1.70

Note: B, unstandardized regression coefficient; CI, confidence interval; β, standardized regression coefficient; VIF, variance inflation factor; SHSE, secondhand smoke exposure. These backward-reduced models are secondary, parsimonious summaries. Because variable selection and coefficient estimation were performed in the same sample, their confidence intervals and p-values do not account for model-selection uncertainty; inference is based on the complete models reported in Tables 2 and 3 of the manuscript.

Table S3. Behavioral-response model: backward-reduced model (secondary summary).

$F(5, 354) = 96.488$, $p < 0.001$; $R^2 = 0.577$; adjusted $R^2 = 0.571$; $N = 360$.

Independent variable	B	95% CI for B	β	*p*-Value	VIF
Age	1.623	(0.687, 2.560)	0.135	0.001	1.31
Gender	3.988	(2.732, 5.244)	0.223	<0.001	1.06
Marital status	−1.521	(−2.868, −0.175)	−0.090	0.027	1.37
Monthly income	−1.661	(−2.391, −0.931)	−0.180	<0.001	1.36
SHSE knowledge score	0.502	(0.439, 0.564)	0.621	<0.001	1.28

Note: B, unstandardized regression coefficient; CI, confidence interval; β, standardized regression coefficient; VIF, variance inflation factor; SHSE, secondhand smoke exposure. These backward-reduced models are secondary, parsimonious summaries. Because variable selection and coefficient estimation were performed in the same sample, their confidence intervals and p-values do not account for model-selection uncertainty; inference is based on the complete models reported in Tables 2 and 3 of the manuscript.

Table S4. Hierarchical multiple linear regression predicting behavioral-response scores among non-smokers ($n = 265$).

Independent variable	Model 1				Model 2			
	B	95% CI for B	β	*p*-Value	B	95% CI for B	β	*p*-Value
Age	2.493	(1.134, 3.852)	0.224	<0.001	1.036	(0.057, 2.014)	0.093	0.038
Gender	4.852	(2.907, 6.797)	0.265	<0.001	3.734	(2.351, 5.118)	0.204	<0.001
Education level	−1.210	(−4.113, 1.692)	−0.054	0.412	−0.611	(−2.667, 1.444)	−0.027	0.559
Employment status	−3.548	(−6.290, −0.806)	−0.180	0.011	−0.903	(−2.871, 1.064)	−0.046	0.367
Marital status	−2.077	(−4.604, 0.450)	−0.114	0.107	−0.873	(−2.668, 0.922)	−0.048	0.339

Independent variable	Model 1				Model 2			
	B	95% CI for B	β	*p*-Value	B	95% CI for B	β	*p*-Value
Number of children	−1.052	(−2.648, 0.544)	−0.085	0.195	0.732	(−0.419, 1.883)	0.059	0.212
Nationality	0.154	(−3.227, 3.534)	0.005	0.929	−0.341	(−2.734, 2.053)	−0.011	0.779
Monthly income	−2.202	(−3.368, −1.035)	−0.242	<0.001	−1.190	(−2.025, −0.356)	−0.131	0.005
SHSE knowledge score	—	—	—	—	0.508	(0.445, 0.570)	0.684	<0.001
**Model summary**								
R^2	0.276				0.639			
Adjusted R^2	0.253				0.626			
ΔR^2	—				0.363			
F (df1, df2)	F(8, 256) = 12.189				F(9, 255) = 50.077			
p-Value for F	<0.001				<0.001			
F-change (df1, df2)	—				F(1, 255) = 256.035			
p-Value for F-change	—				<0.001			

Note: B, unstandardized regression coefficient; CI, confidence interval; β, standardized regression coefficient; SHSE, secondhand smoke exposure; ΔR^2, change in R^2 from Model 1 to Model 2. Model 1 includes the eight prespecified sociodemographic covariates only; Model 2 adds the SHSE knowledge score. Both models were estimated by ordinary least squares in the non-smoker subsample (n = 265; 73.6% of the analytic sample of N = 360). Degrees of freedom for the F statistic are given as (df1, df2). The F-change statistic tests the increment in explained variance attributable to the addition of the SHSE knowledge score in Model 2. Variance inflation factors ranged from 1.04 to 1.81 in Model 2, indicating no problematic multicollinearity.

This subsample analysis is a prespecified sensitivity analysis, reported because protective behavioral responses toward secondhand smoke are conceptually most interpretable among non-smokers. All eight covariates were entered simultaneously; no variable-selection procedure was applied to these models, so the confidence intervals and p-values reported here are not affected by selection uncertainty. The findings are consistent with the full-sample hierarchical model reported in Supplementary Tables S9 and S10, in which the SHSE knowledge score was likewise the strongest correlate of behavioral response.

Table S5. Participants' responses to the secondhand smoke exposure (SHSE) knowledge items (N = 360).

Item	Strongly disagree	Disagree	Don't know	Agree	Strongly agree
Breathing smoke from other people's cigarettes is harmful to one's health	15.0	3.3	4.7	33.3	43.6
SHS contains substances known to be toxic	7.8	9.2	7.5	35.3	40.3
There is no safe level of exposure to SHS	11.9	5.3	12.8	34.2	35.8
Non-smokers can develop lung cancer due to exposure to SHS	6.7	9.4	13.1	36.9	33.6
Exposure to SHS has an immediate effect on the cardiovascular system	12.5	4.4	13.6	37.2	32.2
Risk of coronary heart disease increases with SHS exposure	7.2	9.4	15.8	34.4	33.1
Pregnant women exposed to SHS are more likely to give birth to low-weight babies	12.5	5.3	20.8	33.1	28.3
SHS is associated with sudden infant death syndrome	7.5	10.8	20.0	33.1	28.6
Children exposed to SHS have a higher risk of ear infections	13.9	6.9	18.3	31.9	28.9

Note: SHS, secondhand smoke; SHSE, secondhand smoke exposure. All values are percentages (%) of respondents (N = 360). Rows may not total exactly 100% because of rounding. Items were scored on a five-point Likert scale.

Table S6. Participants' behavioral responses toward secondhand smoke exposure (SHSE) (N = 360).

Item	Strongly disagree	Disagree	Don't know	Agree	Strongly agree
If I am exposed to cigarette smoke from a stranger, I would ask them to put out the cigarette.	15.8	14.4	8.6	33.3	27.8
I normally reduce the duration of conversation with a person who is smoking.	7.5	18.1	9.4	33.9	31.1
I avoid being part of a group of people who are smoking.	13.3	11.4	7.8	32.8	34.7
I avoid talking to someone who is smoking.	9.4	19.4	8.1	34.2	28.9
I try to spend as little time as possible in places where smoking is prevalent.	13.1	10.6	8.9	36.1	31.1
I do not mind chatting with family members while they are smoking.*	42.5	21.4	9.7	19.2	7.2
If I am in a taxi and the driver starts smoking, I would ask them to stop.	13.6	10.3	10.6	32.8	32.8
Visitors are allowed to smoke in my house.*	53.6	13.1	10.6	16.7	6.1
I would not have any objection if someone who is traveling in the same car as me starts to smoke.*	30.3	13.9	20.3	27.5	8.1

Note: SHSE, secondhand smoke exposure. All values are percentages (%) of respondents (N = 360). Rows may not total exactly 100% because of rounding. Items were scored on a five-point Likert scale.

* Reverse-coded tolerance items (shown in italics). These three items were reverse-scored before computing the behavioral-response composite. In the confirmatory factor analysis they showed weak standardized loadings on the Behavior factor (0.22, 0.30, and −0.18, respectively), compared with 0.86–0.95 for the six protective-action items. The Behavior factor had AVE = 0.563 and √AVE = 0.750, which was below the latent inter-factor correlation (r = 0.836); therefore, the Fornell–Larcker criterion was not met. No items were deleted.

Table S7. Bootstrapped mediation analysis: employment status → SHSE knowledge → behavioral response.

Path	B	SE	*p*-Value	95% BCa CI
c (employment → behavior; total effect)	−6.196	—	<0.001	—
a (employment → knowledge)	−7.205	—	<0.001	—
b (knowledge → behavior | employment)	0.534	—	<0.001	—
c′ (employment → behavior; direct effect)	−2.348	—	0.002	—
Indirect effect (a × b)	−3.848	—	—	−5.199, −2.573

Note: B, unstandardized coefficient; SE, standard error; BCa CI, bias-corrected and accelerated bootstrap confidence interval; SHSE, secondhand smoke exposure. The indirect effect accounted for approximately 62% of the total effect, and the significant direct effect indicated partial rather than full mediation. The BCa confidence interval is reported for the indirect effect.

Table S8. Knowledge model: full pre-elimination model.

$F(8, 351) = 18.000$, $p < 0.001$; $R^2 = 0.291$; adjusted $R^2 = 0.275$.

Independent variable	B	95% CI for B	β	*p-Value*	VIF
Age	2.398	(0.811, 3.984)	0.161	0.003	1.46
Gender	−0.632	(−2.699, 1.434)	−0.029	0.548	1.11
Education level	2.059	(−1.069, 5.187)	0.072	0.196	1.53

Employment status	−5.085	(−7.908, −2.263)	−0.219	<0.001	1.88
Marital status	−4.134	(−6.678, −1.591)	−0.198	0.002	1.89
Number of children	−4.059	(−5.751, −2.366)	−0.280	<0.001	1.74
Nationality	1.581	(−2.799, 5.960)	0.032	0.478	1.03
Monthly income	−1.253	(−2.585, 0.080)	−0.110	0.065	1.74

Note: B, unstandardized regression coefficient; CI, confidence interval; β, standardized regression coefficient; VIF, variance inflation factor; SHSE, secondhand smoke exposure.

Table S9. Behavioral-response model 1: full pre-elimination model (without knowledge).

$F(8, 351) = 18.873$, $p < 0.001$; $R^2 = 0.301$; adjusted $R^2 = 0.285$. Step 1 of the hierarchical model (sociodemographic covariates only); N = 360.

Independent variable	B	95% CI for B	*β*	*p-Value*	VIF
Age	2.700	(1.427, 3.973)	0.225	<0.001	1.46
Gender	3.495	(1.836, 5.153)	0.195	<0.001	1.11
Education level	2.462	(−0.048, 4.972)	0.107	0.055	1.53
Employment status	−3.116	(−5.381, −0.851)	−0.166	0.007	1.88
Marital status	−4.188	(−6.229, −2.147)	−0.248	<0.001	1.89
Number of children	−1.264	(−2.622, 0.094)	−0.108	0.068	1.74
Nationality	0.092	(−3.422, 3.606)	0.002	0.959	1.03
Monthly income	−2.451	(−3.520, −1.382)	−0.266	<0.001	1.74

Note: B, unstandardized regression coefficient; CI, confidence interval; β, standardized regression coefficient; VIF, variance inflation factor; SHSE, secondhand smoke exposure.

Table S10. Behavioral-response model 2: full pre-elimination model (with knowledge).

$F(9, 350) = 54.252$, $p < 0.001$; $R^2 = 0.582$; adjusted $R^2 = 0.572$. Step 2 of the hierarchical model (SHSE knowledge score added); $\Delta R^2 = 0.282$; F-change(1, 350) = 236.144, $p < 0.001$; N = 360.

Independent variable	B	95% CI for B	*β*	*p-Value*	VIF
Age	1.479	(0.482, 2.476)	0.123	0.004	1.49
Gender	3.817	(2.533, 5.101)	0.213	<0.001	1.11
Education level	1.413	(−0.534, 3.361)	0.061	0.154	1.54
Employment status	−0.526	(−2.310, 1.258)	−0.028	0.563	1.95
Marital status	−2.082	(−3.684, −0.480)	−0.123	0.011	1.94
Number of children	0.803	(−0.281, 1.887)	0.068	0.146	1.85
Nationality	−0.713	(−3.435, 2.008)	−0.018	0.607	1.03
Monthly income	−1.813	(−2.645, −0.982)	−0.197	<0.001	1.76
SHSE knowledge score	0.509	(0.444, 0.575)	0.630	<0.001	1.41

Note: B, unstandardized regression coefficient; CI, confidence interval; β, standardized regression coefficient; VIF, variance inflation factor; SHSE, secondhand smoke exposure.
